# Editorial: Oxidative stress related to cellular metabolism in lung health and diseases

**DOI:** 10.3389/fphar.2022.1015423

**Published:** 2022-11-03

**Authors:** Karuppusamy Arunachalam, Krishnan Anand, Subramanian Palanisamy, Vikas Anathy

**Affiliations:** ^1^ Key Laboratory of Economic Plants and Biotechnology and the Yunnan Key Laboratory for Wild Plant Resources, Kunming Institute of Botany, Chinese Academy of Sciences, Kunming, China; ^2^ Southeast Asia Biodiversity Research Institute, Chinese Academy of Sciences, Naypyidaw, Myanmar; ^3^ Centro de Estudos em Células Tronco, Terapia Celular e Genética Toxicológica (CeTroGen), Programa de Pós-graduação em Saúde e Desenvolvimento na Região Centro-Oeste, Faculdade de Medicina Dr. Hélio Mandetta (FAMED), Universidade Federal de Mato Grosso do Sul (UFMS), Campo Grande, Mato Grosso do Sul, Brazil; ^4^ Department of Chemical Pathology, School of Pathology, Faculty of Health Sciences, University of the Free State, Bloemfontein, South Africa; ^5^ Department of Marine Food Science and Technology, Gangneung-Wonju National University, Gangneung, Gangwon, South Korea; ^6^ East Coast Life Sciences Institute, Gangneung-Wonju National University, Gangneung, Gangwon, South Korea; ^7^ Department of Pathology, University of Vermont, Burlington, VT, United States

**Keywords:** reactive oxygen species, oxidative stress, lung disease, respiratory illness, drug toxicity and therapy

Cellular metabolism is constantly exposed to oxidants that are produced either endogenously (e.g., by mitochondrial electron transport during respiration or the activation of phagocytes) or exogenously (e.g., by air pollutants and cigarette smoke) (Wilson et al., 2002; Sharifi-Rad et al., 2020). ROS-mediated damage to the lungs is facilitated by the high oxygen concentration in the lungs as well as their large surface area and blood supply. Due to an imbalance of oxidants/antioxidants favoring oxidants, oxidative stress causes the oxidation of proteins, DNA, and lipids, and causes secondary metabolic ROS production in cells (Sies, 1991; Park et al., 2009). The role of oxidative stress in lung diseases such as asthma, bronchial asthma (BA), chronic obstructive pulmonary disease (COPD), acute respiratory distress syndrome (ARDS), acute lung injury, and pulmonary fibrosis has now been established (Park et al., 2009).

According to Adeloye et al. (2022), COPD was the third leading cause of death among people aged 30–79 in 2019, affecting approximately 391.9 million people worldwide. In 2015, 0.4 million people died from asthma, making it the most prevalent chronic childhood medical condition (Maurer, 2016; Soriano et al., 2017; The Global Asthma Report, 2018; Mitra et al., 2021). In children under 5 years of age, pneumonia is the most common cause of death. Millions of people are affected by pneumonia each year. The World Health Organization estimates that over 10 million people worldwide die from *tuberculosis* (TB) every year, making it the most common infectious disease that causes death (WHO, 2016). Globally, lung cancer incidence, mortality, and prevalence are increasing. According to GLOBOCAN estimates, 2.09 million new cases (11.6%) of lung cancer and 1.76 million deaths (18.4%) occur each year (Bade and Cruz, 2020).Worldwide, 545 million people suffered from chronic respiratory diseases in 2017 (Labaki, and Han, 2020).

Respiratory and lung disease medicine is one of the least developed therapeutic fields, with few innovative pharmaceutical products under development. The reasons for this may be a lack of interest among pharmaceutical firms (despite the prevalence of respiratory disorders in the general population), a lack of funding for fundamental research, inadequate and nonpredictive animal models, or a lack of useful biomarkers. Natural chemicals such as epinephrine, herbal anticholinergics, and adrenal corticosteroids are the most commonly used medications nowadays and perhaps in the future. Unfortunately, novel medications have been unsuccessful in treating asthma (leukotriene receptor antagonists and anti-IgE) and COPD (roflumilast) (Gross and Barnes, 2017).

In recent years, ethnopharmacological studies focused on the bioactivities of natural products [(Aescin (*Aesculus hippocastanum* L.), Baicalin (*Scutellaria baicalensis* Georgi), Bavachinin (*Psoralea corylifolia* L.), Capsaicin (Chilli), Carnosic acid (Rosemary), Celastrol (*Tripterygium wilfordii* Hook.f.), Corylifol A (*Psoralea corylifolia* L.), Curcumin (*Curcuma longa* L.), Emodin (*Polygonum cuspidatum* Siebold & Zucc.), Eriodictyol (*Dracocephalum rupestre* Hance), Ginkgetin (*Ginkgo biloba* L.), Glycyrrhizin (*Glycyrrhiza glabra* L.), Jubanine G (*Ziziphus jujuba* Mill.), Kaempferol (Tea), Luteolin (peppermint), Naringenin (Citrus fruits), Resveratrol (Grapes), Salinomycin (*Streptomyces albus*), and Vanillin (*Vanilla planifolia* Andrews)] against metabolic disorders in lung diseases, since many non-natural, synthetic drugs cause various side effects. Several natural remedies have been shown to possess remarkable bioactivities and are considered to be excellent treatments for lung disease (Rahman et al., 2022). The compounds present in these natural remedies function as antioxidants thereby reducing the risk of asthma, COPD, ALI, lung fibrosis, and lung cancer. However, many natural functional metabolites remain unexplored, and the mechanisms related to their beneficial properties remain unclear. In view of the emerging advances in ethnopharmacology for drug discovery, Frontiers in Pharmacology is presenting a Research Topic entitled *Oxidative stress related to cellular metabolism in lung disease and health* to exhibit recent advances concerning natural products and oxidative stress in inflammatory pulmonary diseases, and possible mechanisms of action. A total of eight original research articles and one review article are included in this Research Topic, having generated 6,709 views and 1,358 downloads to date.

This Research Topic includes a review article by Cui et al., which summarizes current research on the molecular mechanisms of ER stress in pulmonary disease, offering several likely therapeutic causes of pulmonary disease including pulmonary fibrosis, pulmonary infection, asthma, COPD, and lung cancer. In addition, the latest interventions to target ER stress and ROS in pulmonary diseases were discussed.

In the medical management of patients with chronic obstructive pulmonary disease (COPD), herbal supplements exhibiting antioxidant and anti-inflammatory properties have proven to be incredibly effective. In the study by Ghobadi et al., COPD patients in the intervention group received crocin supplementation (30 mg/day for 12 weeks), derived from Saffron (*Crocus sativus* L.). Intervention with crocin for 12 weeks decreased the serum levels of TOS and NF-κB and increased TAOC. Crocin supplementation appears to improve inflammatory conditions and establish an oxidant/antioxidant balance in COPD patients.

In a similar study, Liu et al. evaluated the efficacy of Number 2 Feibi Recipe (N2FBR, a traditional Chinese medicine formula) for treating idiopathic pulmonary fibrosis. As a result of N2FBR treatment, interstitial fibrosis and collagen deposition were effectively alleviated, primarily through upregulation of SOD, GSH-Px, and TAOC, and downregulation of MDA. In addition, N2FBR increased the expressions of LC3B, Beclin-1, LAMP1, and TFEB, and downregulated the expressions of p62 and legumain. TEM observations showed that the N2FBR treatment boosted autophagosome production. In addition, the authors revealed the antioxidative stress and proautophagy effects of N2FBR through GSK-3β/mTOR signaling.

Antioxidant products show promise in the treatment and prevention of respiratory tract diseases. Due to its high nutritional value and pharmacological properties such as hypolipidemicity, hypoglycemicity, and antihypertensivity, as well as its antioxidant and anti-inflammatory properties, *Spirulina platensis*, a filamentous cyanobacterium, has been used as a food supplement by humans for many years (Appel et al., 2018). Brito et al. supplemented rats with *S. platensis* at doses of 50 (SG50), 150 (SG150), and 500 mg/kg (SG500) and the nitrite levels, lipid peroxidation, and antioxidant activity were evaluated through biochemical analysis. The authors observed a decrease in lipid peroxidation in the plasma as well as an increase in oxidation inhibition in the trachea and lung for the SG150 and SG500 doses, suggesting enhanced antioxidant activity. Additionally, the antioxidant activity and nitrite levels increased, and the inflammatory responses were modulated, resulting in a decreased contractile response and an increase in relaxation. Fan et al. used C57B6/J mice and human pulmonary microvascular endothelial cells (HPMECs) to investigate the effects of ventilator-induced lung injury (VILI) and Intermedin (IMD), a member of the calcitonin gene-related peptide (CPRP) superfamily. They also evaluated the potential mechanism by investigating anti-apoptotic and antioxidant stress in mice and HPMECs, in this study discovered that IMD decreased VILI as well as suppression of the JAK2/STAT3 signaling pathway was a mediator of the anti-apoptotic and antioxidant stress effects of IMD.

A multidisciplinary approach was used to study the effects of *Thuja occidentalis* L. (TO) homeopathic mother tincture and TO-mediated PDT (TO-PDT) on A549 lung cancer cells using photodynamic therapy (PDT). In cells treated with TO and TO-PDT, morphological changes in the nucleus and cell membrane were evident. A dose-dependent increase in LDH release was observed, as well as a decrease in ATP levels and cell viabilities in these groups, indicating the cytotoxic and antiproliferative properties of TO. At the same doses, TO enhanced anticancer responses when photoactivated in PDT, outperforming TO tincture alone. The results demonstrate that TO can be used as a photosensitizer during PDT to enhance its direct cytotoxic effects on cancer cells Loonat et al.

Evidence suggests that chloroquine (an agonist of bitter taste receptors) improves the cell function of ASM cells, which are involved in asthma. Mice were administered chloroquine or dexamethasone before being introduced to house dust mites (HDMs). According to an *in vitro* study, chloroquine markedly reduced maladaptive changes in the ASM phenotype, combined with a reduction in ROS production. In ASM cells, chloroquine inhibits the ROS-AKT response *via* inhibition of oxidative stress levels and PI3K signaling (LY294002). This may constitute a potential mechanism for restoring the phenotypic imbalance caused by H_2_O_2_ and PI3K inhibition. The findings suggest that chloroquine improves asthmatic airway function by controlling the change in the ASM cell phenotype, providing a new therapeutic profile for airway remodelling Ren et al.

The effects of montelukast and fluticasone on sodium arsenite-induced EMT changes in normal human bronchial cells were investigated. Montelukast was effective in reducing arsenic-induced EMT in human bronchial epithelial cells. Montelukast hindered arsenic-induced cell migration as well as the expressions of extracellular matrix proteins and NF-kB, critical for arsenic-induced EMT, by inhibiting ROS generation and NF-kB activation. In combination with fluticasone, montelukast reversed the inhibitory effects of arsenic on EMT. The purpose of this study was to provide therapeutic strategies and mechanisms for lung epithelia damage caused by arsenic.

Clinical trials have confirmed that the Huashi Baidu Formula (HSBDF) exhibits remarkable clinical efficacy in treating acute lung injury (ALI); it has been approved by the National Medical Products Administration of China. A network pharmacology approach was used to identify the potential regulatory mechanisms of plasma compounds. In order to identify the active compounds, a series of experimental assays was conducted, which included CCK-8, EdU staining, TNF-α, IL-6, MDA, and T-SOD tests, as well as flow cytometry. The pharmacokinetic properties of the active compounds were also predicted. Molecular docking revealed that HSBDF hampered inflammation by inhibiting IL-6R and TNF-α. In the plasma, six key compounds may ameliorate ALI by regulating inflammation and oxidative damage: piceatannol, emodin, aloe-emodin, rhein, luteolin, and quercetin. In addition, the authors found that HSBDF can enhance anti-inflammation and anti-oxidative defenses, as well as decrease cell apoptosis Wang et al.

To summarize, in recent years numerous studies have been performed to analyze the impact of natural products derived from plants and animals. Polyhydroxy compounds such as flavonoids, alkaloids, and terpenoids derived from plants exhibit a variety of *in vitro* and *in vivo* biological effects. These include anti-inflammatory, antiviral, antiplatelet, anti-tumor, anti-allergic, antioxidant, and immunomodulatory effects. Substances with different chemical structures have different biological effects. However, many of these natural drugs do not pass clinical trials due to their toxicities and low bioavailabilities. Acceptable drugs for lung diseases must be screened using pharmacokinetics and pharmacodynamic studies. The literature does not contain many clinical trials using these compounds, and further research is required to confirm their safety and efficacy. In the future, research should focus on the identification and isolation of more effective compounds, their mechanisms of action, formulations, dosage forms, pharmacokinetics, and safety profiles, in order to develop more effective multi-target drugs for respiratory and lung diseases.
